# Deficiency of N-linked glycosylation impairs immune function of B7-H6

**DOI:** 10.3389/fimmu.2023.1255667

**Published:** 2023-11-15

**Authors:** Hanqing Chen, Yang Zhang, Yu Shen, Liang Jiang, Guangbo Zhang, Xueguang Zhang, Yang Xu, Fengqing Fu

**Affiliations:** ^1^ Jiangsu Institute of Clinical Immunology, the First Affiliated Hospital of Soochow University, Suzhou, China; ^2^ Department of Hematology, the First affiliated Hospital of Soochow University, Suzhou, China; ^3^ Department of Respiratory and Critical Medicine, the Affiliated Suzhou Hospital of Nanjing Medical University, Suzhou Municipal Hospital, Gusu School, Nanjing Medical University, Nanjing, China; ^4^ Suzhou Red Cross Blood Center, Suzhou, China; ^5^ National Clinical Research Center for Hematologic Diseases, Jiangsu Institute of Hematology, the First Affiliated Hospital of Soochow University, Suzhou, China

**Keywords:** N-linked glycosylation, B7-H6, NK cell, NKp30, binding affinity

## Abstract

B7-H6 is a novel immune checkpoint molecule that triggers NK cell cytotoxicity, but the role of N-glycosylation in B7-H6 is poorly understood. We here identified the existence of N-glycosylation of B7-H6 in different cell lines and exogenous expression cells by PNGase F digestion and tunicamycin blockage. Subsequently, we demonstrated that B7-H6 contains 6 functional N-linked glycosylation sites by single site mutation and electrophoresis. Phylogenetical and structural analysis revealed that N43 and N208 glycan are conserved in jawed vertebrates and may thus contribute more to the biological functions. We further demonstrated that N43 and N208 glycosylation are essential for B7-H6 to trigger NK cell activation. Mechanistically, we found that N43 and N208 glycan contributed to the stability and membrane expression of B7-H6 protein. Lack of N208 glycosylation led to membrane B7-H6 shedding, while N43 mutation resulted in impaired B7-H6/NKp30 binding affinity. Together, our findings highlight the significance of N-linked glycosylation in B7-H6 biological functions and suggest potential targets for modulating NK cell-mediated immunity.

## 1 Introduction

Natural Killer (NK) cells are the most abundant subset of innate lymphoid cells (ILCs) in peripheral blood, and serve as one of the most important immune surveillance defense lines against virus or other intracellular pathogens-infected cells, senescent cells and tumor cells (1, 2). Distinct from T cells, NK cells activation is not restricted by the major histocompatibility complex (MHC) (3) but is triggered by the signal balance of various molecules with opposing functions, either activating or inhibitory (4). Upon activation, NK cells exhibit potent cytotoxicity by releasing perforin, granzyme and other immune-stimulating cytokines.

B7 homolog 6 (B7-H6), a natural ligand for NKp30 that triggers NK cell cytotoxicity, belongs to B7 costimulatory molecule family and is a type I transmembrane glycoprotein with 454 amino acids (5). Natural cytotoxicity receptor 3 (NCR3), also known as NKp30, is one of four currently discovered NK cell cytotoxicity trigger receptors (including NKp30, NKp44, NKp46, NKp80), and is constitutively expressed in NK cells (6). Upon engagement to its ligand, NKp30 transduces a strong activation signal through immunoreceptor tyrosine‐based activation motif (ITAM)-bearing adaptor proteins CD3ζ (7). Unlike another NKp30 ligand, BAT3 (HLA‐B‐associated transcript 3, also identified as BAG6, BCL2‐associated athanogene 6),which normally acts as an intracellular molecular chaperon (8), B7-H6 is located on the cell surface and upregulated in stressed and tumor cells (5, 9). Previous studies have demonstrated the association of the B7-H6 molecule with a various cancers including glioma (10),neuroblastoma (11), breast cancer (12), lung cancer (13), acute myeloid leukemia (14), lymphoma (15) and cervical carcinoma (16). Additionally, B7-H6 also plays an important role in inflammation (17) and virus-infection (18, 19). Although the importance of B7-H6 is gradually being disclosed, the mechanisms by which B7-H6 is modulated are still poorly understood, especially in the field of post translational modification (PTM).

N-linked glycosylation, one of the most common PTMs of membrane proteins in eukaryotic cells, is the attachment of an oligosaccharide to a nitrogen atom from the amide nitrogen of an asparagine (Asn) residue present as a part of Asn-X-Ser/Thr (X is any amino acid except proline) consensus sequence (20). This type of glycosylation initiates simultaneously with translation progress at endoplasmic reticulum (ER) lumen by transferring a precursor oligosaccharide to certain synthesizing peptides recognized by oligosaccharide transferase (OST). Subsequently, N-glycan trimming proceeds in ER and Golgi body, and forms a complex and heterogeneous oligosaccharides structure of N-glycosylation, and the glycoprotein is further modified by the addition and removal of sugar residues catalyzed by Golgi resident glycosyltransferases (GRGT) (21, 22). It is well known that N-linked glycosylation assists protein folding and works as a part of protein quality control system in eukaryotic cells (23). Additionally, such a process plays an important role in regulating protein stability, subcellular location as well as protein interaction affinity (24). Consequently, N-glycosylation affects numerous aspects of biology including cell communication, adhesion, migration, signaling metabolism, tumor generation and immune response (25).

There is increasing evidence demonstrating the importance of the N-glycosylation in immune checkpoint molecules. PD-L1/PD-1 is one of the most well-studied molecular pairs in B7-CD28 family, both of which are modified with N-glycan (26–28), and new therapy strategies to target the carbohydrate component of these glycoproteins are gradually emerging with promising results (29). N-glycosylation stabilizes PD-L1 protein by preventing degradation through GSK3β-mediated 26S proteasome pathway, thus improving the efficiency of blocking T cell activation (26). Moreover, glycosylation of PD-L1 is necessary for interaction with PD-1 while the specific contribution of each oligosaccharide attached on four Asn residues and detailed mechanisms on atomic level remain unclear (30). It has also been reported that the N-linked glycosylation of PD-L1 significantly interferes with the prognosis and clinical diagnosis of patients in cancer therapy (27). In terms of PD-1, Fut-8 catalyzes core-fucosylation of N49 and N74 glycans, thus promoting expression of PD-1 protein on cell-surface (31). Similar to these findings, it has been well established that N-glycosylation is crucial for other immune checkpoint molecules. B7-H3 and B7-H4 are stabilized by N-glycosylation in triple negative breast cancer, resulting in immune suppression of T cells (32, 33). Meanwhile, a high proportion of carbohydrate conjugates consisting of α-galactose and fucose was detected in B7-H3 expressed by oral epithelium carcinoma cell compared with normal cells, these aberrant sugar residues can better interact with DC-SIGN and Langerin expressed by dendritic cells or macrophages (34).

As an essential member of B7 molecule family, previous studies have shown that B7-H6 protein has six predicted N-glycosylation sites (35), and PNGase F treatment shifts electrophoresis bands (36), while the function of glycosylation of B7-H6 protein has not been further investigated. Here, we focused on the immune function of B7-H6 glycosylation and identified the key modification sites that impair NK cell cytotoxicity. Specifically, the deficiency of N43 and N208 glycosylation led to significant decrease of NK cell killing through distinct mechanisms. On one hand, N43 glycosylation enhanced B7-H6/NKp30 interaction affinity, as well as stabilized B7-H6 protein itself; On the other hand, N208 glycosylation stabilized the membrane B7-H6 protein from being shed as soluble form. These findings not only expand our knowledge regarding the function of N-linked glycosylation in B7-H6 molecule, but also provide us with new perspectives for cancer immunotherapy.

## 2 Materials and methods

### 2.1 Cell culture

HEK293T, U87, NB4, HL-60, HCT116 cells were maintained in high glucose Dubbecco’s modified Eagle’s medium (DMEM, Gibco) supplied with 10% fetal bovine serum (FBS, EVERY GREEN), 10 µM β-mercaptoethanol and 1% L-glutamine (Beyotime). THP-1, U937, HEL, Daudi, Raji, DB, Jurkat, MM1.1 cells were maintained in RPMI 1640 medium (Gibco) supplemented with 10% FBS, 10 µM β-mercaptoethanol and 1% L-glutamine. K562 and SKM-1 were maintained in DMEM/F12 medium (Gibco) supplied with 10% FBS, 10 µM β-mercaptoethanol and 1% L-glutamine. NK-92 cells were cultured in α-minimal essential medium (α-MEM, GIBCO) adding 15% FBS, 0.2 mM inositol, 0.02 mM folic acid, 10 µM β-mercaptoethanol, 1% L-glutamine and 20 ng/mL rhIL-2 (Peprotech). These cells were all kept in humidified incubators with 5% CO_2_ at 37°C. Specifically, EXP293 cells for eukaryotic expression system were maintained in serum-free medium BI-293 (Biointron Biological Inc) supplied with 1% L-glutamine and kept vibrating at 120 RPM under humified atmosphere containing 8% CO_2_ at 37°C. Cells were dispersed with 0.25% trypsin (Beyotime) containing 5 mM EDTA if necessary before passaging.

### 2.2 Western blot

Cell lysates were prepared by adding 2×sample buffer directly, followed by ultrasonic sonication and boiling if not specially mentioned. Glycosidase treated cell lysates were prepared by TETN lysis buffer (20 mM tris-HCl pH 7.4, 5 mM EDTA, 150 mM NaCl, 1% triton-X100, 10% glycerol) incubating at 4°C with gentle shaking for 10 min. Then supernatant was collected by centrifuging at 12,000 g at 4°C for 20 min. After glycosidase catalysis, protein samples were mixed 4:1 with 5×sample buffer and boiled for gel loading. WB analysis was performed according to standard protocol by separating the protein sample with SDS-PAGE, followed by wet transfer to a PVDF membrane (Merck Millipore). A mouse anti-human B7-H6 monoclonal antibody (Suzhou Bright Scistar Antibody Biotech), rabbit anti-FLAG antibody (Cell Signaling Technology) and mouse anti-human ACTB monoclonal antibody (Abclonal) were used at corresponding recommended concentrations incubating overnight at 4°C. Finally, HRP-conjugated goat anti-rabbit or goat anti-mouse H+L chain multiclonal antibodies (Multi Sciences) were incubated at RT for an hour at a concentration of 1:10 000. The blots were visualized with an ECL detection kit (NCM biotech) and imaged by a ChemiScope system (Model No.6300). Image processing and analysis were done with photoshop software.

### 2.3 Plasmid and lentivirus construction

The ORF region of B7-H6 gene was inserted into pIRES2-EGFP and pSIN-EF1α vectors by restriction cloning (enzyme from NEB and TAKARA) or in-fusion cloning (Clontech) for transient or stable expression respectively. Site directed mutation was generated by overlap extension method to create B7-H6 expression vectors with single or multiple glycosylation site N→Q mutation. All vector sequences were confirmed by sanger sequencing before use. Transfection were performed by using lipofectamine 3000 (Invitrogen) as a ratio to plasmid with 3-4:1 following manufacturer’s recommendation. Lentiviral vectors were constructed by co-transfection pSIN-EF1α and two envelop plasmids pMD2.G, pxPAX2 to HEK293T at a ratio of 2.5:1:1. Lentiviral vectors containing supernatant was obtained 48 h after transfection. B7-H6 KO HEK293T and U87 cells were infected with lentivirus and 8 µg/ml polybrene, 4 µg/ml puromycin was added for selection stable clone for one week. Successful transduction was all confirmed by flowcytometry respectively.

### 2.4 CRISPR induced gene knockout

Regarding the knockout of B7-H6, we performed CRISPR-Cas9 assay using three plasmids, one encoding the Cas9 gene, one encoding guide RNAs and the other one encoding a puromycin-resistance gene, respectively. The guide RNAs used to target B7-H6 were listed in supplementary. Briefly, HEK293T and U87 cells were co-transfected with the four plasmids with Lipofectamine 3000 (Invitrogen). Puromycin (5 μg/mL) (Sigma-Aldrich) was used subsequently to kill untransfected cells, and then a single cell was picked to form a colony. Successful double-strand edited clones were identified by sanger sequencing and flowcytometry analysis (**Figure S2**).

### 2.5 Soluble protein extraction

Protein sample from cell culture supernatant were prepared by TCA/acetone precipitation method. In general, serum-free culture cell supernatant was harvested 24 h after medium refreshing when cell reached about 90% confluence. Equal volume of 20% (w/w) trichloroacetic acid solution was mixed with samples and precipitated at -20°C overnight. Precipitation was separated by 12,000 g centrifugation and washed with acetone twice. Finally, sediment was dissolved with TETN buffer containing 1% (w/w) SDS and boiled for 20 min. The supernatant containing soluble part of denatured protein was retained for WB analysis after centrifuge.

### 2.6 Protein expression and purification

Before transfection, 0.22 µm filtered 200 μg expression vector (pcDNA3.4 was used for expression and insert gene structures was shown in **Figure S3**) was diluted to 1.25 ml with BI-293 medium and pre-incubated with 1.25 ml linear 40 kDa polyethyleneimine (lPEI, polyscience) at a 1:3 DNA:lPEI (w/w) ratio diluted in BI-293 as well for 20 min. EXP293 cells were centrifuged for 5 min at 90 g, and were resuspended at the concentration of 40×10^6^/mL in 2.5 mL of BI-293 medium. Subsequently, cells were mixed with DNA-PEI mixture in an empty flask and were incubated in high density at 20×10^6^/mL on a shaker for 2–4 h at 37°C. Afterwards, up to 50 mL BI-293 medium,2 mM sodium butyrate, 1% F68 (Gibco) and 3% serum free feeder (Gibco) were added to medium, and feeder was replenished every other day. Cells were discarded after 6 days by centrifugation for 30 min at 4000g at 4°C. The supernatant was filtered through a 0.2 µm filter and loaded onto a pre-equilibrated 1 mL protein A column or Ni column using an SCG FPLC system respectively (Sepure Instruments). Fc-tagged protein was eluted with 25 mM glycine-HCl pH 3.0, and neutralized with 1/10 volume 1M Tris-HCl pH 7.4 and His-tagged protein was eluted with 500 mM imidazole. Final concentration was performed using ultrafiltration centrifuge tube (10k or 25k MW, Millipore) at 6000g, 4°C and change buffer to PBS. Protein concentration was determined by Microspectrophotometer (biorad).

### 2.7 ELISA and ELISA based interaction detection

B7-H6/NKp30 binding affinity was detected with ELISA based method. Briefly, 1µg/ml wild type or glycosylation-site-mutated hFc-tagged B7-H6 protein was coated on a polystyrene ELISA plate. After 37°C overnight PNGase F (NEB) treatment and 3 times of washing, 5% BSA was used for non-specific blocking. The indicated concentration ladder was diluted using pre-incubated 5 µg/ml rhNKp30-his protein and HRP conjugated anti-his monoclonal antibody (Cwbio), and then added into plate and incubated at 4°C for another 2 h. An equivalent HRP conjugated anti-his monoclonal antibody pre-incubated without NKp30 protein was used as a negative control. Development was done used TMB substrate (ebioscience) and final absorbance value was acquired on a microplate reader (Thermo) at OD_450_. Notice, PNGase F digestion was performed on the plate and Triton X-100 was added in digestion buffer to avoid plate adsorption. Denatured enzyme was obtained by incubating at 70 °C for 10 min and sufficient digestion was confirmed by western blot.

### 2.8 Flow cytometry

Antibody staining was performed following a standard method. A purified mouse IgG1 (BioLegend) and the PE-conjugated anti-mouse IgG antibodies (Multi Sciences) were used as isotype controls for indirect staining, while a PE-conjugated mouse IgG1 antibody (BioLegend) was used for direct staining. A mouse anti-human PE-conjugated B7-H6 antibodies (R&D Systems) were used at the concentration of 1 μL/test and incubated at 4°C for 20 min. NKp30 binding was measured by previously incubating NKp30-his recombination protein and PE conjugated anti-his antibody (BioLegend) at 5 μg/mL for 1 hour at 4°C, then staining cells with NKp30-antibody complex at 1 μg/mL at 4°C for 20 min. Fluorescent staining was analyzed with an FC500 flow cytometer (Beckman Coulter) and FlowJo software (Tree Star.Inc).

### 2.9 NK cell cytotoxicity assay

NK cell cytotoxicity was measured by calcein-AM labeling (37). Briefly, HEK293T cells that stablely expressed control vector, wild type or glycosylation site mutated B7-H6 protein and HCT-116 cells transfected with same plasmids were trypsinized and labeled with 5 μM calcein-AM (Beyotime) in HBSS by incubating at 37°C for 20 min. Cells were then washed twice and seeded into V-shape 96 wells plate at 10^4^ cells/well with adding organic anion transporter inhibitor probenecid (MCE) at 2.5 mM. Indicated number of NK-92 cells were mixed with each target cells in triplicate and incubated for 6 h respectively. Afterwards, maximum release wells were obtained by adding Triton X-100 to break down target cells. Supernatant was collected and the fluorescence (Excitation, 490 nm, Emission, 530 nm) was measure on a fluorescent microplate reader (TECAN). Cell cytotoxicity was calculated by the following formula:


$$\text{\% cytotoxicity}=\frac{experimental\;\text{fluorescent}\;-\;NK\;cell\;\text{fluorescent}\;-\;target\;cell\;\text{fluorescent}\;}{target\;cell\;maximum\;\text{fluorescent}\;-\;medium\;background}\times 100\%.$$

### 2.10 NK cell activation and CD107a turnover measurement

HEK293T cells that stablely expressed control vector, wild type or glycosylation site mutated B7-H6 protein were trypsinized and seeded into 96 wells plate at 5×10^4^ cells/well. Two folds of NK-92 cells were mixed with each type of HEK293T cell in triplicat respectively. 1µL PE conjugated anti-CD107a antibody (Biolegend) was added into each well and incubated for 8 h at 37°C. Afterwards, cells were collected and stained with PE-cy7 conjugated anti-CD69 antibody (BD). After washing for 3 times, the cells were resuspended, and the fluorescence signal was analyzed by flow cytometry.

### 2.11 CHX-chase assay

HEK293T and U87 cells that stablely expressed B7-H6 were trypsinized and then seeded into 24 wells plate. After 24h incubation, 2.5 µg/ml N-linked glycosylation inhibitor tunicamycin (TM, Selleck) was added to cells and 5 µg/ml *de novo* protein synthesis inhibitor cycloheximide (CHX, Selleck) was added after another 24h. Cells were harvested at 0, 2, 4, 8, 12, 24 h time points respectively for immunoblotting assay. For glycosylation site mutation samples, cells that stablely expressed wild type or N43Q, N208Q B7-H6 protein were seeded into multi-well plates and treated with CHX only, and harvested at indicated time points. The relative intensities of bands were normalized to β-actin, and 0 h samples were considered 100%. Fitting curves were plotted according to the equation $\text{y}=\text{N}\times {\left(\frac{1}{2}\right)}^{\frac{x}{t}}$ .

### 2.12 Phylogenetic analysis and amino acid conservation scoring

The whole length of the deduced amino acid sequence of B7-H6 protein from 82 species were aligned using MEGA X software. A Neighbor-joining tree was built using bootstrap method with 2000 replications. Amino acid conservation was calculated by AACon service contained in JABAWS software (38).

### 2.13 Statistics

Statistics analysis was performed using Graphpad Prism 9 software.

## 3 Results

### 3.1 Human B7-H6 protein is highly glycosylated with N-linked glycans

**Figure 1:**
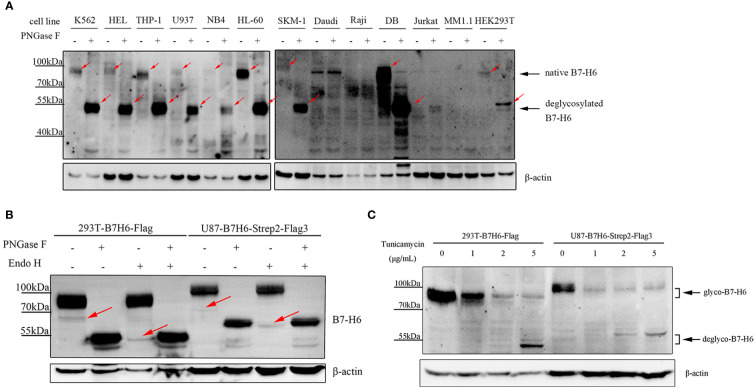
Human B7-H6 protein is modified with N-linked glycosylation. **(A)** protein samples of the indicated cell lines were treated with or without PNGase F overnight and loaded for WB analysis. Red arrows indicated specific signals of B7-H6. **(B)** B7-H6 knockout 293T and U87 were exogenously overexpressed with B7-H6, and protein sample of these two cells were obtained for PNGase F or Endo H glycosidase digestion. WB was performed to analyze band shifting. Red arrows indicated weak signals of B7-H6. **(C)** B7-H6 overexpressed 293T and U87 cells were treated with the indicated concentration of tunicamycin for 48 h, band shift was analyzed by WB.

B7-H6 protein consists of 454 amino acids and has a molecular weight of about 50.8 kDa as predicted. However, consistent with a previous study (36), B7-H6 displayed a heterogeneous smear of bands ranging from 80 – 95 kDa on SDS-PAGE electrophoresis in 9 of 13 different cell lines (including K562, HEL, THP-1, U937, NB4, HL-60, SKM-1, DB and HEK293T) (**Figure 1A**). Undetectable of B7-H6 in Daudi and Raji was confirmed by flowcytometry due to unspecific bands on blots (**Figure S1**). To confirm that the heterogeneous bands were caused by N-linked glycosylation, we treated these samples with PNGase F, which removes all N-linked oligosaccharides from glycoproteins and decreases the apparent molecular weight to a predict one if the molecule contains only N-glycans modification. As a result, B7-H6 in all 9 cell lines showed a predict molecular weight of about 51 kDa after PNGase F treatment (**Figure 1A**). Additionally, we knocked out (KO) endogenous B7-H6 in 293T and U87 cells with CRISPR-Cas9 system, and the knockout validation was achieved by Sanger sequencing. Since membrane B7-H6 could not be detected in U87 cells by flow cytometry analysis, the validation of this part could only be performed in 293T (**Figure S2**). Then, cells overexpressing the exogenous B7-H6 with C-terminus FLAG tag or 2×Strep-3×FLAG tag were generated by infecting two KO cell lines separately using a lentiviral vector. 293T and U87 used in the following experiments represent these two gene-edited engineered cells. Next, both PNGase F and Endo H glycosidase were used to digest the glycoprotein and our results showed that PNGase F digestion shifted the whole B7-H6 band to predicted MW, while Endo H could only catalyze the minor 65 kDa band to 51 kDa (**Figure 1B**). Thus, it can be inferred that the majority of B7-H6 proteins with an approximate 90 kDa MW bear a complex type N-linked glycosylation that is insensitive to Endo H digestion, though a small amount of high mannose type oligosaccharides presents at ~65 kDa could be easily digested by Endo H. In parallel, tunicamycin, an antibiotic that inhibits GlcNAc phosphotransferase (GPT) catalyzing the transfer of N-acetylglucosamine-1-phosphate from UDP-N-acetylglucosamine to dolichol phosphate in the first step of glycoprotein synthesis, was used to specifically block N-linked glycosylation in B7-H6 overexpressed cell lines (i.e., 293T and U87). Consistently, in both cell lines, tunicamycin treatment led to a remarkable shift of the B7-H6 band to a predicted MW (**Figure 1C**). These data collectively suggest that B7-H6 is a glycoprotein modified with N-linked glycosylation.

### 3.2 B7-H6 protein has six functional N-glycosylation motifs

**Figure 2:**
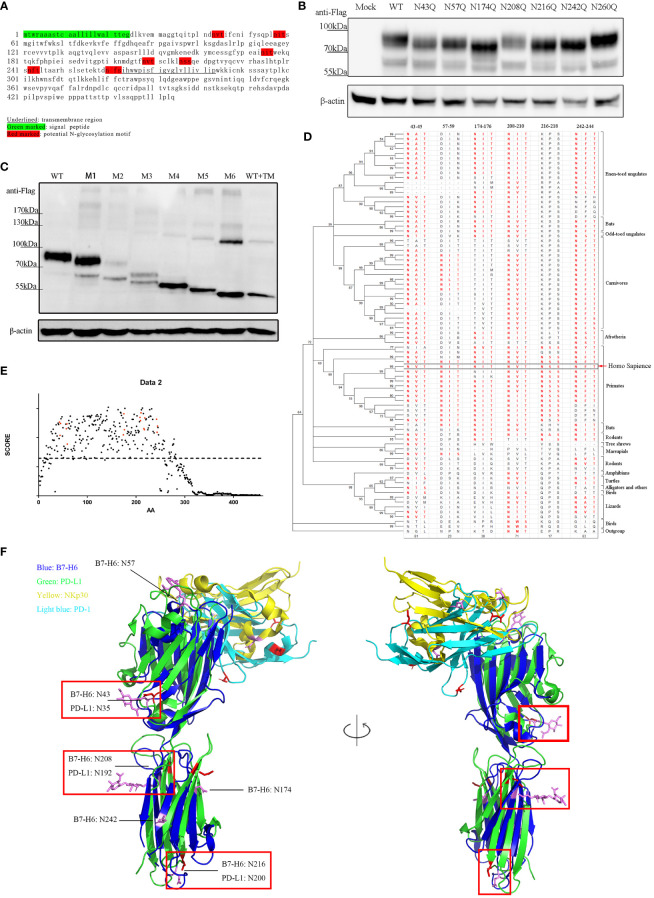
B7-H6 protein contains 6 functional N-glycosylation sites. **(A)** sequence analysis of B7-H6 protein amino acid. Red blocks indicate six N-X-S/T motifs. **(B)** 293T cells were transfected with B7-H6 plasmid of wild type or single N→Q mutation at the indicated position. Cell lysates were obtained 48 h after transfection and WB was performed with anti-Flag tag antibody. **(C)** 293T cells were transfected with B7-H6 plasmid of wild type or multiple N→Q mutation from N terminus. Cell lysates were obtained 48 h after transfection and WB was performed with anti-Flag tag antibody. Wild type plasmid transfected 293T cells were treated with tunicamycin for 24 h before harvesting, and were used as a total deglycosylated control. **(D)** phylogenetic tree of B7-H6 protein across jawed vertebrates was generated based on 82 species. Amino acid sequences were obtained from NCBI. Sequence homology to human B7-H6 N-X-S/T motifs was listed and red fonts indicated potential glycosylation ability. **(E)** Amino acid conservation was calculated by AACON method. Red dot indicated amino acid presented in N-X-S/T motifs, dotted line indicated an average score of all B7-H6 amino acids. **(F)** Alignment of B7-H6/NKp30 complex and PD-L1/PD-1 complex. Crystal structures were downloaded from PDB. Red rectangles highlighted overlaid glycosylation sites of B7-H6 and PD-L1.

To identify the N-glycosylation motifs in B7-H6 protein, we predicted N-glycosylation motifs by amino acid sequence analysis. We showed that there were seven N-X-S/T motifs in the extracellular domain of human B7-H6 protein. Among them, N^43^-V-T (N43 motif) and N^57^-I-T (N57 motif) were located in IgV domain, which could directly interact with NKp30, N^174^-I-T (N174 motif), N^208^-V-T (N208 motif), N^216^-S-S (N216 motif), N^242^-F-T (N242 motif) were located in IgC domain, while N^260^-F-S (N260 motif) was exactly adjacent to the transmembrane domain (**Figure 2A**). To test whether these motifs were functional, we constructed single N→Q mutation expression plasmids at each site and transduced them into B7-H6 KO 293T cells. By electrophoresis detection, B7-H6 protein with single N-linked glycosylation motif mutation sites except N260Q displayed a faster migration rate in SDS-PAGE compared to wild type control. The data indicated that among all seven N-glycosylation motifs, N43, N57, N174, N208, N216 and N242 motifs were functionally involved in B7-H6 glycosylation (**Figure 2B**). Notably, N174Q and N216Q bands migrated faster than the other four mutations, suggesting that N174/N216 might bear an oligosaccharide with more complex sugar components. We further generated another series of plasmids containing an increased amount of mutation sites from one to six (M1, N43Q; M2, N43Q+N57Q; M3, N43Q+N57Q+N174Q; M4, N43Q+N57Q+N174Q+N208Q; M5, N43Q+N57Q+N174Q+N208Q+N216Q; M6, N43Q+N57Q+N174Q+N208Q+N216Q+N242Q). Consequently, the electrophoresis rate of B7-H6 protein gradually increased with the successive number of mutant sites, while the band consisting of all six mutation sites shifted to the same position as the tunicamycin treated wild type plasmid (**Figure 2C**). Thus, we concluded that human B7-H6 protein contains six effective N-glycosylation sites.

Conservation analysis can provide us with a powerful tool to deduce whether a certain glycosylation site is important. The more conserved motif is in evolution, the more likely the oligosaccharide attached to that site is critical to the biological function of the protein. We downloaded B7-H6 amino acid sequences of 82 species of jawed vertebrates from the NCBI (National Center of Biotechnology Information) database. Amino acid conservation scoring indicated that all six motifs were conserved across species compared to an average score of all B7-H6 amino acid (dotted line) (**Figure 2E**). By Motif searching accompanied with phylogenetic tree construction, we found that N43/N208/N242 were conserved in almost all jawed vertebrates, N57 was conserved in primaries and present in some carnivores, N174 was present in almost all placental mammals except rodents and carnivores, while N216 was only conserved among primates (**Figure 2D**). As B7-H6 shared the highest similarity with the B7 family molecules PD-L1 and B7-H3 (39), we found that the 3D structure of B7-H6 was more similar to PD-L1 than B7-H3 (RMSD PD-L1 3.283 vs. B7-H3 3.494). N-linked glycosylation sites on the alignment layout of B7-H6 (3PV6) and PD-L1 (3BIK) were labeled, among which, obviously, four glycosylation sites of B7-H6 at N43/N174/N208/N216 corresponded to N35/N192/N200/N219 in PD-L1 molecule, respectively (**Figure 2F**). Combining the results of sequence analysis and structure alignment, we hypothesized that N43 and N208 glycosylation sites might contribute more to the biological function of B7-H6.

### 3.3 Defective N-glycosylation of B7-H6 impairs NK cell cytotoxicity

**Figure 3:**
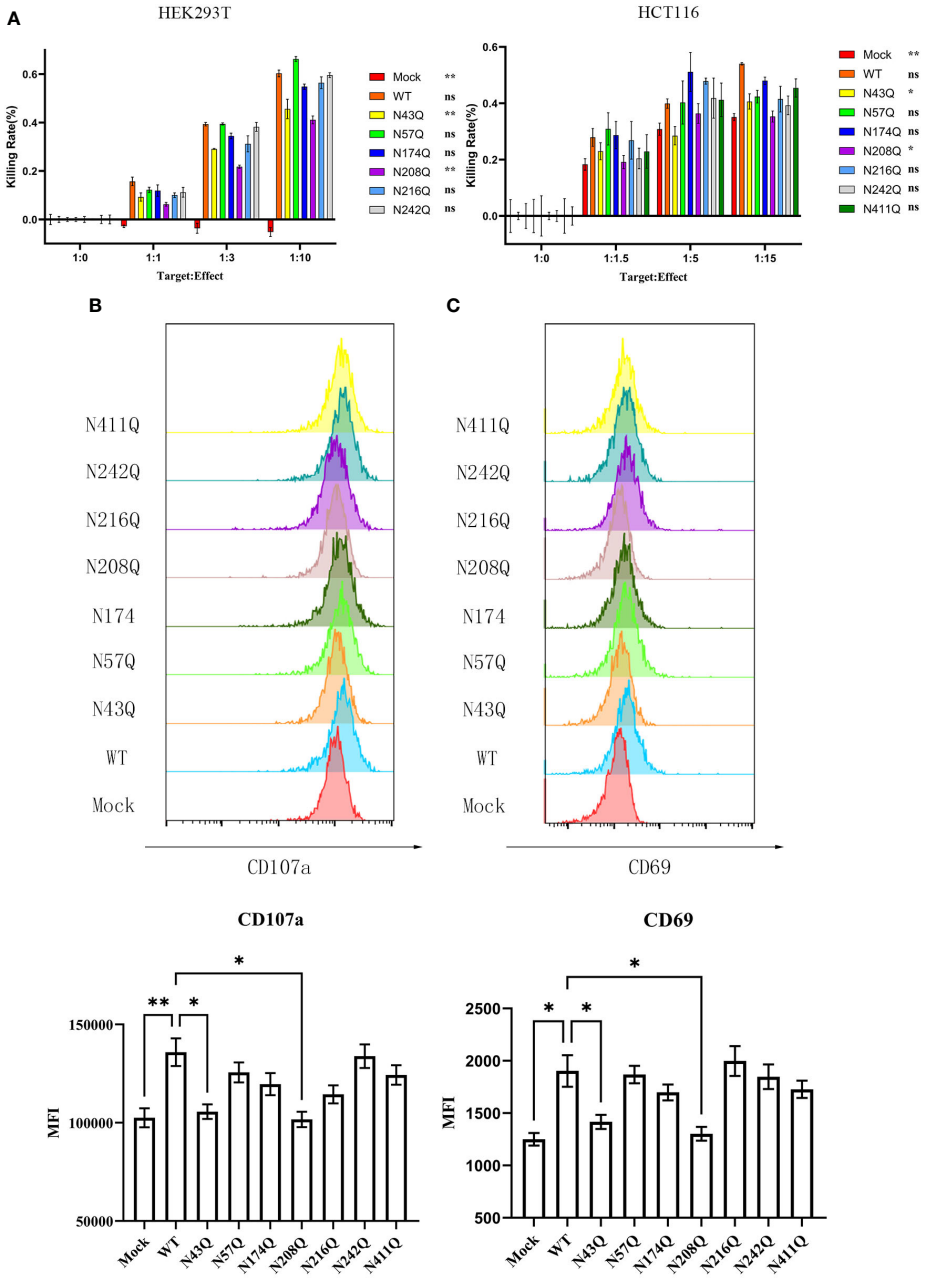
Defective N-glycosylation of B7-H6 impairs NK cell cytotoxicity. **(A)** NK cell cytotoxicity was measured with calcein-AM labeling method, data were collected with 3 replications. HEK293T (left) and HCT-116 (right) were used as target cells respectively. Significance was calculated by two-way ANOVA method comparing WT to other groups. **(B)** CD107a turnover assay was performed 8 hours post coculture of 293T and NK92. MFI was shown below. **(C)** NK cell activation marker CD69 was detected after coculture at the same time. Data were shown as mean ±SD , and significance was analyzed by two-way ANOVA. ns, no significance; *, p<0.05; **, p<0.01.

To address whether glycosylation affects the function of B7-H6, we investigated the cytotoxicity of NK cell using NK92, a NK lymphoma cell line that maintains the capacity to kill target cells mainly through NKp30 pathway, when cocultured with 293T cells overexpressed wild type or single site mutated B7-H6 protein. Interestingly, calcein-AM release assay revealed that glycosylation deficiency at N43 and N208 of B7-H6 significantly impaired the cytotoxicity of NK cell, while mutations at other glycosylation sites had no effect on the effectiveness of NK cell killing (**Figure 3A**, left). Due to the heterogeneity of glycosylation in different cells, we also repeated the killing assay in HCT-116 cells, which naturally do not have detectable B7-H6 on membrane (**Figure S1D**), transfected with same array of plasmids. Data indicated consistent decrease of killing rate of N43 and N208 mutation (**Figure 3A**, right). Meanwhile, NK cell degranulation and activation were measured by CD107a turnover assay and CD69 labeling, respectively. The data showed that, consistent with the results of cytotoxicity assay, NK92 activation and degranulation induced by target cell were almost completely lost when a single N→Q mutation occurred at N43 or N208 in B7-H6 molecule. However, no significant changes were observed for other glycosylation site mutations (**Figures 3B, C**). Thus, we uncovered that N43 and N208 glycosylation are key sites that determine the biological function of B7-H6 in triggering NK cell killing.

### 3.4 Deficiency of N-linked glycosylation decreases B7-H6 protein membrane location and stability

**Figure 4:**
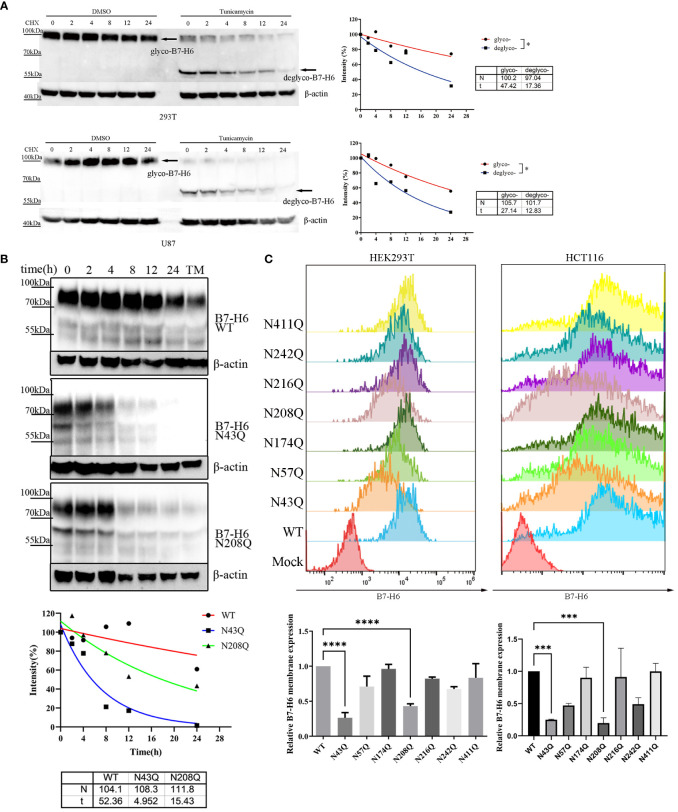
N43 and N208 glycosylation regulates B7-H6 protein membrane location and stability. **(A)** the stability of tunicamycin induced N-glycosylation deficiency of B7-H6 protein was measured by WB. 293T or U87 overexpressed B7-H6 were treated with tunicamycin or DMSO were harvested after the indicated period of CHX treatment. The grey intensity of images was analyzed and data were fitted to the equation $\text{y}=\text{N}\times {\left(\frac{1}{2}\right)}^{\frac{x}{t}}$ . **(B)** 293T cells stably expressing wild type, N43Q or N208Q were applied to CHX-chase assay. The grey intensity of images was analyzed and data were fitted to the equation $\text{y}=\text{N}\times {\left(\frac{1}{2}\right)}^{\frac{x}{t}}$ . **(C)** membrane B7-H6 expression of transfected HEK293T (left) and HCT-116 (right) was measured by flow cytometry, MFI was analyzed by one-way ANOVA. Data were shown as mean ±SD . *, p<0.05; ***, p<0.005; ****, p<0.001.

N-linked glycosylation contributes significantly to protein stability. Incorrectly glycosylated protein will be catabolized through ubiquitin-dependent or ER-related degradation pathways, leading to downregulation of protein abundance and resulting in dysfunction of certain proteins. In the case of B7-H6, we verified this assumption by treating overexpressed 293T or U87 cell with N-linked glycosylation inhibitor tunicamycin (TM) in combination with the protein synthesis inhibitor cycloheximide (CHX). Our data indicated that the half-life of B7-H6 was reduced from 47.42 h to 17.36 h in 293T cells and from 27.14 h to 12.83 h in U87 cells, respectively (**Figure 4A**). CHX chasing assay was also used to identify whether the glycosylation of N43 and N208 motifs, which determine the biological function of B7-H6 in triggering NK cell killing, is responsible for B7-H6 protein stability. As expected, either deficiency of glycosylation at N43 or N208 shortened the half-life of B7-H6 protein almost 3 folds, from about 52 h to 5 h with N43 mutation or 15 h with N208 mutation, respectively (**Figure 4B**).

Considering that only the membrane form of B7-H6 could interact with receptor NKp30 and trigger NK cell response, we measured the abundance of membrane bound B7-H6 protein bearing single mutation of N-X-S/T motif by flowcytometry. Consistent with our hypothesis, fluorescence intensity strikingly faded when HEK293T (**Figure 4C**, left) and HCT-116 (**Figure 4C**, right) cells were transfected by plasmid with N→Q mutation in N43 or N208 motifs compared to wild type control.

**Figure 5:**
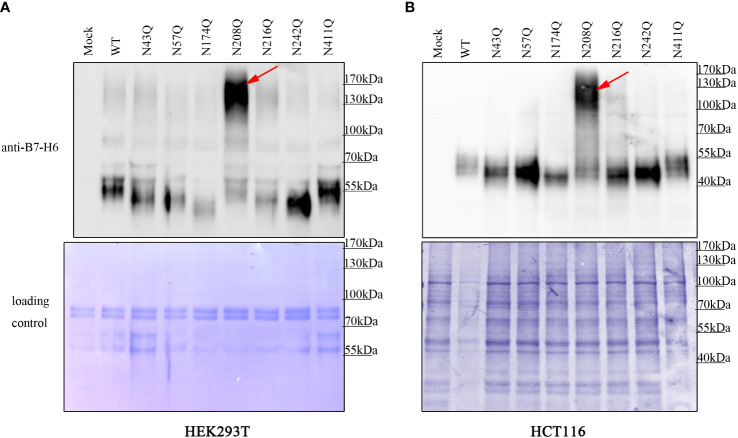
deficient of N208 glycosylation leads to shedding of membrane B7-H6. Supernatant of HEK293T **(A)** cells stably expressing or HCT-116 cells **(B)** transient expressing wild type or single site mutated B7-H6 were collected after 24h incubation. Soluble B7-H6 were precipitated with TCA/acetone method, and analyzed by WB. Red arrow indicated a specific band of sB7-H6 protein generated only with N208Q mutation. Loading control was prepared by Coomassie brilliant blue staining of PVDF membrane.

Although it was clear that the deficiency of glycan at N43 and N208 led to reduction of membrane bound B7-H6, there was still a gap between the different amounts of membrane B7-H6 reduction and the result of inactivation of NK cell caused by the mutation in N43 or N208 motif. Thus, we questioned whether additional mechanism was involved in this process. Interestingly, we found that B7-H6 concentration contained in cell supernatant with N208Q mutation increased dramatically by western blot (WB) and an outstanding band could be observed at 130-170 kDa in both HEK293T cells and HCT-116 cells (**Figures 5A, B**). Combining with the fact that soluble B7-H6 can block NKp30 by working as a decoy, these data suggested that the lack of N208 glycan may not only led to decrease of membrane bound B7-H6 abundance, but also resulted in the shedding of B7-H6 protein releasing into supernatant and subsequently inhibiting NK cell activation.

Together, we identified N43 and N208 as the predominate glycosylation site responsible for B7-H6 stability and membrane location.

### 3.5 N-glycosylation regulates affinity of NKp30/B7-H6 interaction

**Figure 6:**
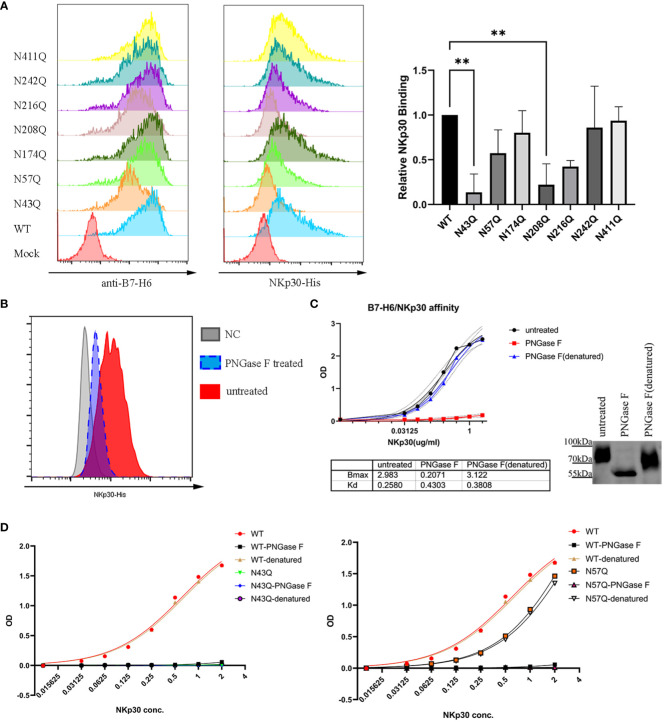
N43 and N57 glycosylation alter B7-H6/NKp30 binding affinity. **(A)** 293T cells stably expressing wild type or single-site mutated B7-H6 were analyzed for B7-H6 expression and NKp30 binding ability by flow cytometry. Relative MFI is shown in **(B)** and analyzed by one-way ANOVA. **(C)** rhB7-H6-hFc recombinant protein was coated and treated with PNGase F, denatured PNGase F or nothing. NKp30 binding ability was detected with an ELISA-based method, data were fitted to the equation of $\text{y}=\frac{x\times Bmax}{Kd+x}$ . Right panel indicated a parallel reaction without coating to the plate. Samples were then analyzed by WB. **(D)** purified wild type, N43Q (left) or N57Q (right) rhB7-H6 recombinant protein were applied to measure NKp30 binding ability with pretreatment of PNGase F or denatured PNGase F or nothing. Data were fitted to the equation of $\text{y}=\frac{x\times Bmax}{Kd+x}$ . Data of D were obtained from three replicates. Data were shown as mean ± SD. **, p<0.01.

To test whether the two motifs N43 and N57 which located in IgV domain of B7-H6 affect the interaction between B7-H6 and NKp30, we first conducted a crude measurement by flow cytometry staining in the transfected cells containing single glycosylation site mutation with B7-H6 antibody and his-tagged NKp30, respectively. The relative fluorescence intensity (MFI normalized to wild type) of N43Q and N208Q stained with NKp30 decreased much more than the corresponding decrease of B7-H6 expression (**Figures 6A, B**). Considering the fact that only N43 and N57 glycosylation sites were located at receptor binding domain, we assumed that mutation at N43Q might lead to a decrease of NKp30 interaction affinity. In order to test this hypothesis, we first purified B7-H6-hFc protein with a eukaryotic expression system. With an ELISA based assay, we treated plate pre-coated with recombinant B7-H6 protein with PNGase F to release the oligosaccharides or heat-denatured PNGase F as a control. A sufficient catalysis of glycosidase and glycan-free protein acquisition was confirmed by WB (**Figure 6C**, right). To our surprise, glycosidase incubation led to almost completely loss of B7-H6/NKp30 binding (**Figure 6C**, left). To eliminate the possibility that glycosidase interfered with the correct interaction of B7-H6 and NKp30, we subsequently purified other two B7-H6-hFc protein with N43Q or N57Q mutation and the same ELISA based affinity detection method was performed for interaction measurement. A dramatical loss of binding ability was also observed when N43 motif was mutated (**Figure 6D**, left). Meanwhile, a significant decrease of binding affinity could also be observed when N57 motif was mutated (**Figure 6D**, right). Additionally, upon PNGase F treatment, wild type, N43Q and N57Q all showed negative signals while denatured PNGase F treatment had no effect at all. Thus, we found that N43 glycosylation greatly affected binding affinity between B7-H6 and NKp30, and N57 glycan also enhanced the binding affinity of B7-H6/NKp30 complex.

## 4 Discussion

In this study, we identified that B7-H6 protein was N-glycosylated at six sites functionally by inducing mutations at each potential modification motif with an N→Q. Among all six N-glycosylation sites, deficiency of N43 or N208 strikingly impairs biological function of B7-H6 resulting in shortening of protein half-life, decreased membrane localization, increased shedding and weakening of B7-H6/NKp30 interactions. According to these findings, we highlighted that N-glycosylation is an indispensable modification for B7-H6 immune function.

N-linked glycosylation is one of the most common post-translational modification of membrane protein. Many immune checkpoint molecules belonging to the B7-CD28 family such as PD-L1/PD-1 molecule pair have been shown to carry N-linked oligosaccharide, which have a great impact on the immune function of modified molecule (40). Although previous study have shown that B7-H6 is predicted to be modified with N-glycan with six potential sites (35), Eva Schlecker and colleagues found that PNGase F treatment of B7-H6 resulted in electrophoresis bands shift to predicted molecular weight in BxPC3 (36). Ondrej Skorepa et al. reported their MS data showing five N-glycosylation sites in addition to N242 in HEK293 cells (35). In this study, we confirmed all six N-glycosylation sites by single site mutation following SDS-PAGE and there were no more undetected modification sites, because the molecule weight of B7-H6 bearing all six sites mutation was consistent with tunicamycin treated one. N242 glycan was not detected in Ondrej Skorepa’s group, which may be attributed to the recombinant protein used only covered amino acid 1 to 245, by contrast, we constructed plasmids using full-length of B7-H6 protein. Additionally, N260 site is also a N-X-S/T motif in which glycosylation was not detected according to our data, and we deduced that N260 may be too close to transmembrane domain that glycotransferases could not contact with it physically due to steric hindrance.

To address the conservation of N-X-S/T motif in B7-H6, we not only drew phylogenetic tree according to DNA sequence obtained from 82 jawed vertebrates, but also compared the three-dimension structure between B7-H6 and other homologs. Although B7-H6 and B7-H3 share the greatest similarity in amino acid sequence and structure (5), the position of attached N-glycan is completely different (**Figure S4C**). PD-L1 is another molecule that shares the highest similarity with B7-H6, meanwhile their four glycosylation sites are close (**Figure 2F**). These data suggested that the same glycotransferases may contribute to the two molecules, and similar mechanism may be involved in the biofunctional changes of N-glycosylation. Combining the results of sequence and structure analysis, N43 and N208 glycosylation were the most likely candidates to contribute greatly to the biological functions of B7-H6.

We subsequently evaluated the significance of B7-H6 biofunction bearing single site mutation at each glycosylation site by using NK-92 cells as an effector cell. Consistent results that loss of N43 or N208 glycosylation impaired B7-H6 bio-activity, were observed in NK cell killing assay, CD107a turn over assay and NK cell activation.

To investigate the detailed mechanism of deficiency of glycosylation leading to impaired B7-H6 biofunction, we first detected the membrane B7-H6 by flowcytometry, as only a membrane bound type could contact with NK cell expressed NKp30 protein and trigger NK cell cytotoxicity. Meanwhile, it makes sense that an abnormally glycosylated protein would undergo degradation or impair the ability of membrane localization, for which some evidence has been observed previously (26, 41, 42). According to our data, membrane abundance of B7-H6 was impaired by N43 or N208 glycosylation site mutation and CHX chasing assay indicated stability decay of B7-H6 protein, as predicted. However, membrane B7-H6 decay may contribute partly to reduction of NK cell cytotoxicity, there might be other mechanisms involved in this interaction. We next tested the culture supernatant of cells with mutated B7-H6. To our surprise, a specific band of B7-H6 appeared only in N208Q with 130-170 kDa, which is equivalent to 3 times the native B7-H6 ECD, but the mechanism of its generation remains unclear. Furthermore, we also noticed that small amount of 130-170 kDa band was exist in wild type supernatant, and confirmed that band was not generated by denatured precipitation (**Figure S4**). We concentrated supernatant from WT B7-H6 expressed by 293T cells by ultrafiltration and digested with or without PNGase F. Then supernatant and cell pellets were analyzed with WB. The digested main 55 kDa bands shifted to 35 kDa, while 130-170 kDa band shifted to about 70 kDa. Additionally, B7-H6 in supernatant lacks the C-terminus Flag-tag confirmed it is a soluble form instead of membrane bond form presenting in extracellular vesicles. As the soluble B7-H6 in N208Q cells increased dramatically, and it was reported that sB7-H6 could be generated by MMPs shedding in different cancer cell lines (36), we hypothesized that the N208 glycosylation protects B7-H6 protein from exposure to certain MMPs and further maintains the membrane bound condition for NK cell recognition. Additionally, sB7-H6 could compete with NKp30 binding to membrane bound B7-H6 according to the literature (17, 36), and a potentially covalent bonded trimer structure might considerably strengthen the binding affinity, which contributed to the cytotoxicity loss of NK cells as one of the factors.

Although the mechanism of N208 glycan deficient induced B7-H6 dysfunction was briefly demonstrated, we considered that other mechanisms were involved in N43 glycan deficiency due to the gap between N43Q induced B7-H6 decay and loss of NK cell cytotoxicity. With a crude NKp30 binding assay based on flowcytometry previously, we further confirmed this hypothesis by testing purified rhB7-H6 protein and PNGase F. Contrary to Skorepa’s (35) data, they observed that digestion of simple glycan type B7-H6 by Endo F1 did not alter its affinity to NKp30 monomer. Endo F1 digestion leaves a single GlcNAc unit at each glycosylation site, distinct to PNGase F digestion and point mutation completely wipe out the oligosaccharides. Additionally, the expression AA length of B7-H6 and the oligomerization status of NKp30 were different from ours. The binding affinity of receptor-ligand pairs modulated by N-glycan has been found in many molecules, and Li (30) tested interaction between immune receptors and ligands by a classical ELISA-based method, the results of which showed that almost half of the tested molecule pairs exhibited impaired binding capability upon PNGase F treatment. To further rule out the possibility that remaining PNGase F interfered with the outcomes, we obtained rhB7-H6 protein bearing N43Q or N57Q mutation, respectively. Our data demonstrated that deficient of N43 glycan led to an almost complete loss of NKp30 binding ability, while deficient of N57 glycan impaired it. Although the evidence is overwhelming, it is still hard to explain the result perfectly. According to the crystal structure, N43 is located on the ligand binding domain of B7-H6, but not adjacent to the binding interface, suggesting that it is impossible for N43 oligosaccharides directly contact with NKp30 geometrically. To our knowledge, it is not a unique case that a glycan interferes with receptor-ligand binding without direct interaction. IL-7/IL-7R is another case reported by (43), although the certain glycosylation site which contributes to the binding affinity remains unclear. PD-L1 exhibits the same property that N-glycans are responsible for binding affinity enhancement, but no glycans can physically interact with PD-1 molecules (30) (**Figure S5A**). As well as TIGIT/PVR complex, author (44) described N101Q of TIGIT strikingly suppresses the interaction towards PVR. But a structure analysis showed N101 glycan also lay far away from the binding interface (**Figure S5A**). Another consideration is that glycan could adjust the fine structure of ligand binding domain, forcing it to maintain a conformation that is well aligned with the ligand. Although previously shown in glycopeptides, glycan can alter conformation preference of the peptide backbone near the glycosylation site (45, 46), it is unlikely that glycan induced significant protein conformation according to a dynamic computation study based on PDB sourced crystal structure (47). Our dynamic simulations of N43 or N57 deficient B7-H6 protein showed no significant conformational changes especially at NKp30 biding interface either (**Figure S5B**). Thus, the contribution of distant N-glycan to receptor/ligand binding is not unique, the special detailed mechanism involved remain to be elucidated.

In summary, we demonstrated that B7-H6 is a N-linked glycosylation modified protein bearing 6 functional glycosylation sites. Among them, N43 and N208 glycan were highlighted because they are not only conserved in various species but also contribute to biological function of B7-H6——to trigger NK cell cytotoxicity. Furthermore, we elucidated that N43 and N208 glycan affected B7-H6 protein stability, and N43 glycosylation enhanced B7-H6/NKp30 interaction, and N208 glycosylation prevented the generation of a specific type sB7-H6 protein. In this study, we expounded the significance of N-linked glycosylation modifications of B7-H6 protein and provided a detailed framework for understanding the function of B7-H6 protein.

## Data availability statement

The original contributions presented in the study are included in the article/**Supplementary Material**, further inquiries can be directed to the corresponding authors.

## Ethics statement

Ethical approval was not required for the studies on humans in accordance with the local legislation and institutional requirements because only commercially available established cell lines were used.

## Author contributions

HC: Conceptualization, Data curation, Formal analysis, Funding acquisition, Investigation, Methodology, Validation, Writing – original draft. YZ: Data curation, Investigation, Methodology. YS: Data curation, Investigation, Methodology. LJ: Funding acquisition, Resources. GZ: Funding acquisition, Resources. XZ: Project administration, Writing – review & editing. YX: Supervision, Writing – review & editing. FF: Conceptualization, Data curation, Resources, Project administration, Supervision, Writing – review & editing.
