# Supplementary material for: Deficiency of N-linked glycosylation impairs immune function of B7-H6
Source: Front Immunol. 2023 Nov 15;14:1255667. doi: 10.3389/fimmu.2023.1255667 (PMC10684670; doi:10.3389/fimmu.2023.1255667)
Supplement: Supplementary file 1 [file DataSheet_1.docx]

Supplement material


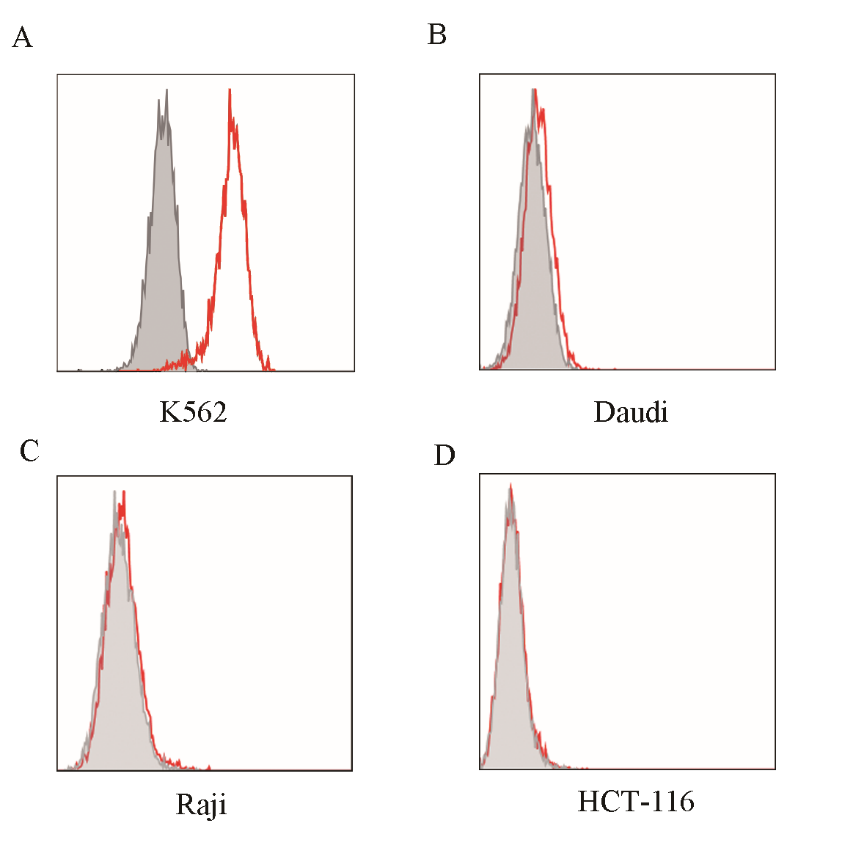


Figure S1. Expression of B7-H6 in different cell lines. B7H6 expression on K562(A, positive control), Daudi(B), Raji(C) and HCT-116(D) cells were measured by flow cytometry.


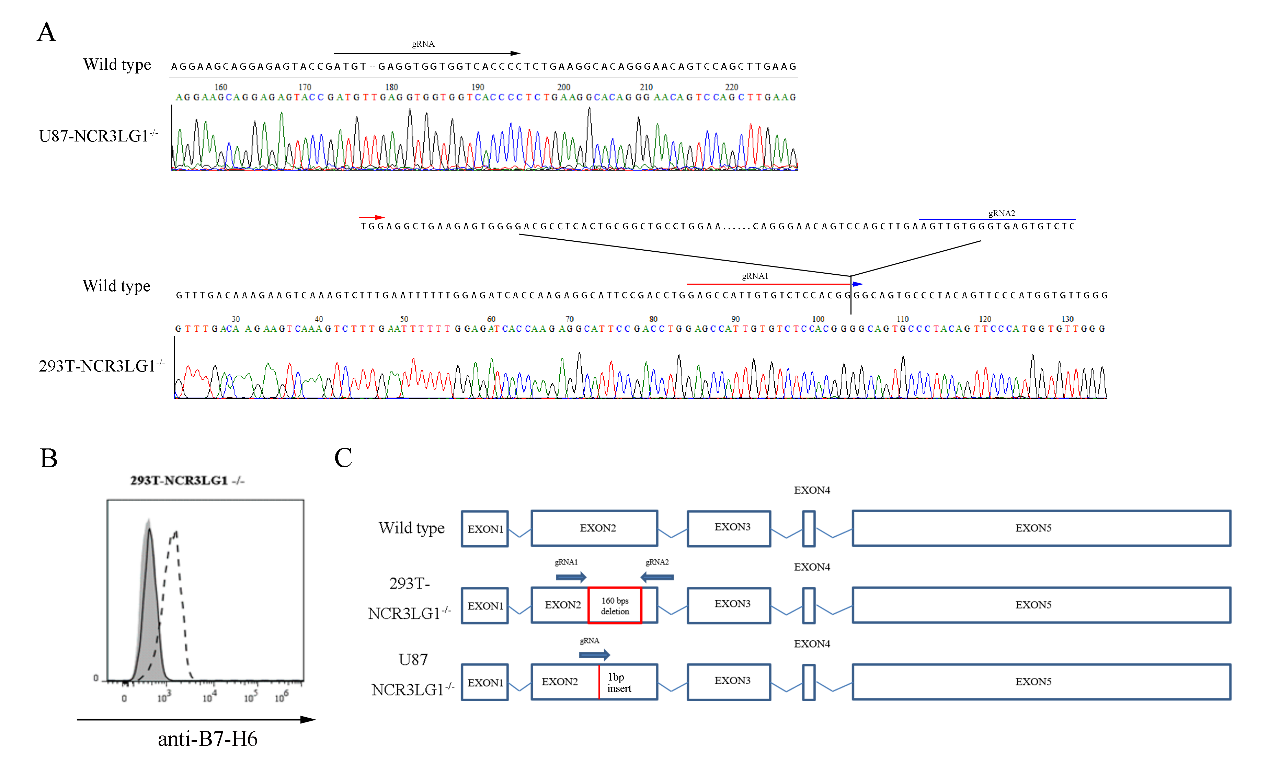


Figure S2. Construction of B7-H6 knockout 293T and U87 cell lines. A. Sanger sequencing results of B7-H6 knockout cell lines were shown with wild type reference sequence. B. knockout of B7-H6 in 293T cell line was confirmed by flowcytometry, black line represented isotype control, dot line represented wild type cells. C. the designing strategy of gRNAs was shown.


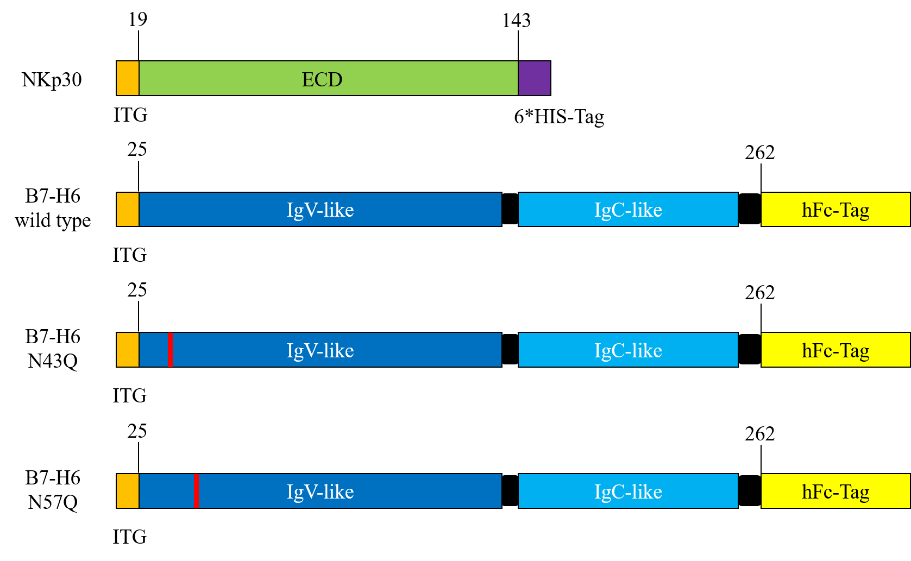


Figure S3. The recombinant proteins used in this study were constructed as shown. 19 – 143 amino acids of NKp30 were linked to 6*His-Tag, while 25 – 262 amino acids of B7-H6 were fused with human IgG Fc fragment. N43Q and N57Q mutations were introduced in B7-H6 N43Q and B7-H6 N57Q proteins respectively.


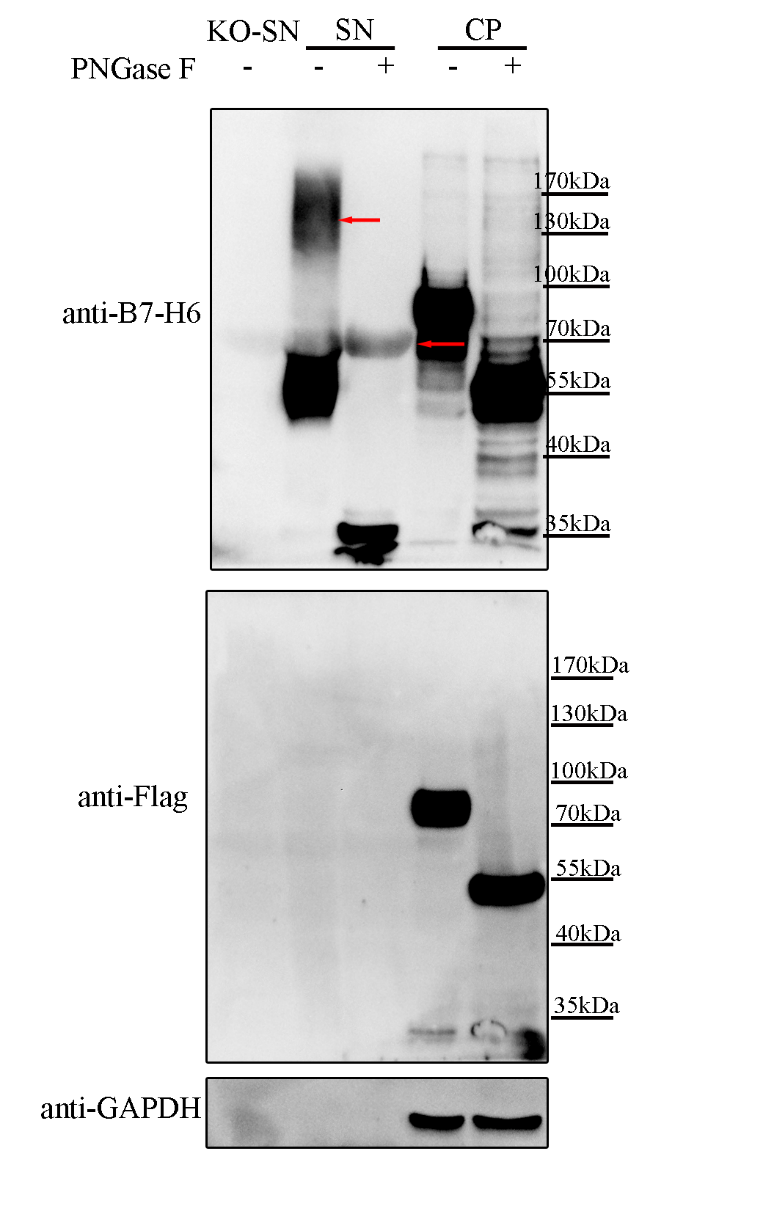


Figure S4. Supernatant from B7-H6-KO and B7-H6 overexpressed 293T cells were obtained after 24 h incubation, and then concentrated with ultrafiltration. Concentrated B7-H6 overexpressed supernatant and cell pellets sample were then treated with or without PNGase F and loading for WB analysis.


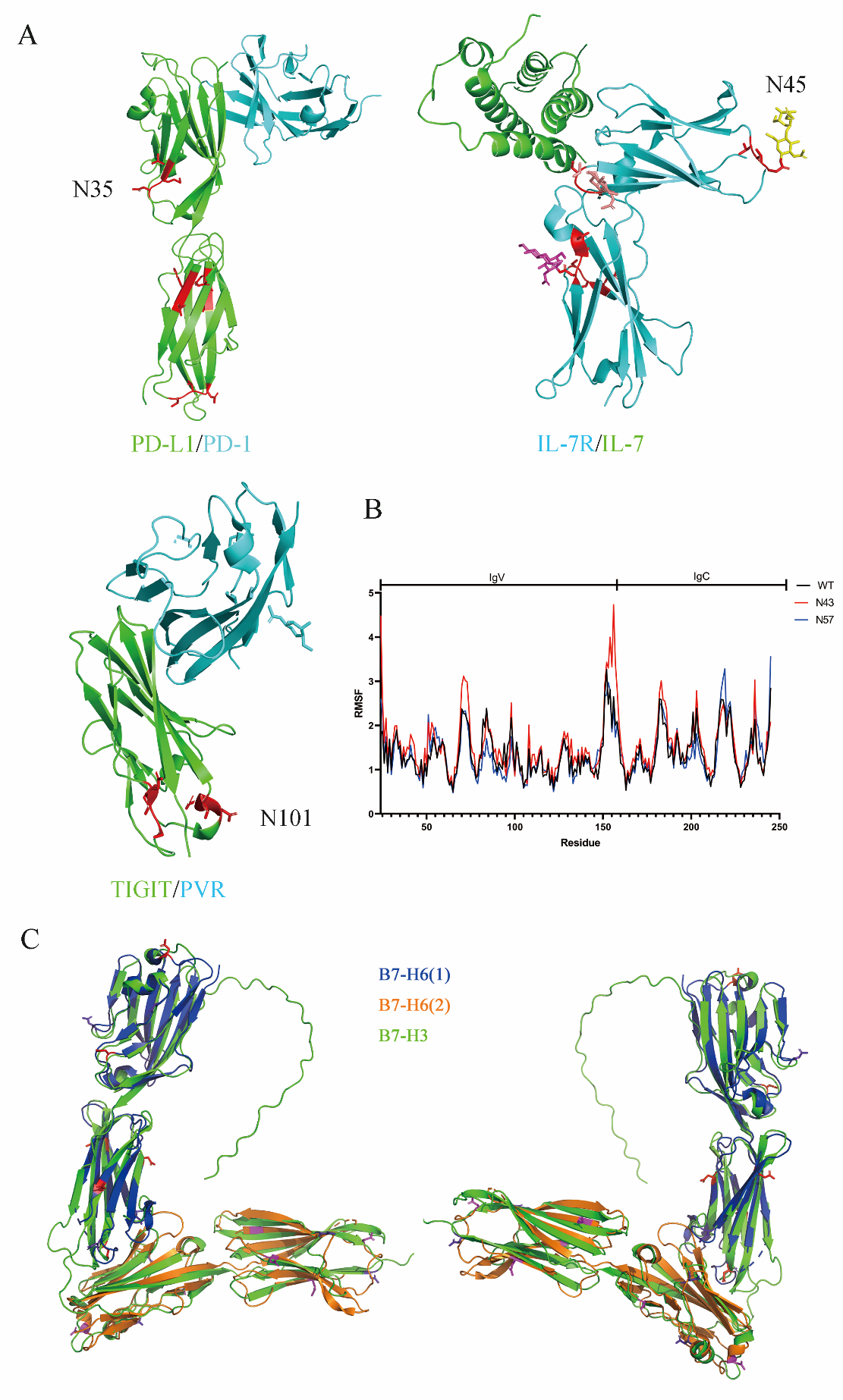


Figure S5. A. crystal structures of PD-L1/PD-1(3BKI), IL7R/IL7(3DI3) and TIGIT/PVR(3UDW) complex were shown. Glycosylation sites of PD-L1, IL-7R and TIGIT were marked red. B. wild type or N43, N57 glycan deficient B7-H6(3BKI) proteins were performed dynamic simulation, RMSF of each residue was plotted. C. crystal structure of B7-H6(3BKI) and B7-H3(AF-Q5ZPR3-F1-model_v4) were shown. B7-H6 was aligned to IgV1-IgC1 and IgV2-IgC2 respectively and glycosylation sites of each molecule were highlighted with different colors.
